# Beverage Consumption among U.S. Children Aged 0–24 Months: National Health and Nutrition Examination Survey (NHANES)

**DOI:** 10.3390/nu9030264

**Published:** 2017-03-13

**Authors:** Carley A. Grimes, Ewa A. Szymlek-Gay, Theresa A. Nicklas

**Affiliations:** 1 Deakin University, Institute for Physical Activity and Nutrition Research, School of Exercise and Nutrition Sciences, Locked Bag 20000, Waurn Ponds, Geelong VIC 3000, Australia; carley.grimes@deakin.edu.au (C.A.G.); ewa.szymlekgay@deakin.edu.au (E.A.S.-G.); 2 Children’s Nutrition Research Center, Baylor College of Medicine, 1100 Bates Ave, Houston, TX 77030, USA

**Keywords:** NHANES, infant, toddler, beverage intake, water intake

## Abstract

Data on beverage consumption patterns in early life are limited. The aim of this study was to describe beverage consumption by sociodemographic characteristics, along with water intake and sources of water among U.S. children aged 0–24 months. Data from 2740 children in the 2005–2012 NHANES were analysed. Food intake was determined via one 24-h dietary recall. Beverages were categorised according to What We Eat In America groups. Poverty–Income ratio was used to define household income. During infancy (0–5.9 months and 6–11.9 months) infant formulas were the most commonly consumed beverage, 74.1% and 78.6% of children consuming, respectively. Comparatively fewer children, 41.6% and 24.3%, consumed breast milk. In toddlers (12–24 months), the most commonly consumed beverages were plain milk (83.6% of children consuming), water (68.6%), 100% fruit juice (51.8%) and sweetened beverages (31.2%). Non-Hispanic black and Mexican-American children were more likely to consume sweetened beverages, 100% fruit juice and infant formula than Non-Hispanic white children. Children from lower income households were more likely to consume sweetened beverages and 100% fruit juice and less likely to consume breast milk than children from higher income households. Total water intake increased with age and the contribution of water from food and beverage sources was ~20% and ~80% for all children, respectively. Disparities in beverage consumption by race/ethnicity and income level are apparent in early life.

## 1. Introduction

Beverages are an important source of energy and micronutrients during the first two years of life [1,2]. Over time, beverage consumption patterns among U.S. children 0–5 years have shifted [3]. In 2001–2006 fewer children drank milk and more children drank 100% fruit juice, compared to earlier periods of 1976–1980; 1988–1994. Although there was no change in the consumption of fruit drinks and soft drinks during this period, the consumption of these beverages was relatively common, consumed by about a third of all children [3]. The American Academy of Pediatrics recommends infants drink breastmilk, or if this is not possible, formula for the first year of life and the introduction of 100% fruit juice should be avoided. If 100% fruit juice is introduced this should be after 6–9 months and limited to no more than 4–6 ounces per day (118–177 mL/day). The introduction of sweetened beverages should also be avoided in the first two years of life, and toddlers should be encouraged to drink water and milk [4].

There are limited data on beverage intake among U.S. infants and toddlers. Earlier reports come from the 1994–1996, 1998 Continuing Survey of Food Intakes by Individuals (CSFII) [5], the 2002 [2,6] and 2008 [7] Feeding Infants and Toddlers Study (FITS) and trend analysis of NHANES data spanning up to 2006 [3]. Some of these reports have excluded participants who were breastfed [3,5]. It is important to understand beverage habits in early life, as this is when food preferences are developed [8]. Furthermore, there is some evidence to suggest that food and beverage habits may track from infancy into early childhood [9,10,11]. The primary purpose of this paper was to describe beverage consumption among U.S. infants and toddlers by sex, age group, race/ethnicity, and poverty–income ratio. As beverages contribute substantially (about 80%) [12] to daily water intakes we also report on water intake from beverage categories and food sources (i.e., moisture in food) as well as total daily water intake. Information on total water intake and sources of water in the diet is particularly relevant for this age group. Infants are susceptible to dehydration because they have a large surface area to volume ratio, which leads to greater water loss, limited ability to excrete renal solutes and inability to communicate thirst [12]. Whilst national data on water intake among children aged 4–12 years have been documented [13], no recent information concerning infants and toddlers is available. The findings from this study will provide information on beverage consumption during the first two years of life and can be used to inform the U.S. 2020 Dietary Guidelines for Infants and Toddlers [14]. In addition, understanding sociodemographic differences in beverage patterns in early life is necessary to identify groups at risk of poor beverage choices and support the development of targeted strategies to improve infant feeding practices.

## 2. Materials and Methods

### 2.1. Study Design and Participants

This study used data from the U.S. 2005–2012 National Health and Nutrition Examination Survey (NHANES) [15]. The NHANES is a cross-sectional survey which utilizes a complex, multistage, probability sampling design. Approval for the study was granted by the National Center for Health Statistics (NCHS) ethics review board [16] and parental/guardian consent was obtained for all participants. Further details of the methodology are reported elsewhere [17]. This analysis includes participants aged from birth to 23.9 months. A total of 2857 children agreed to participate, this analysis includes those children who completed a 24-h dietary recall which was deemed as reliable by NCHS (*n* = 2740). Data from four survey cycles (2005–2006, 2007–2008, 2009–2010, and 2011–2012) were combined. As this was a secondary data analysis which lacked personal identifiers, this study did not require additional institutional review.

### 2.2. Measures

The study child’s proxy self-reported information on the age, sex and race/ethnic group of the child. Race/ethnic group was defined according to the NHANES categories and included non-Hispanic white (Non-HW), non-Hispanic black (Non-HB), Mexican-American (MA), Non-Hispanic Asian, other Hispanic or other race/mixed race. Due to small numbers in the Non-Hispanic Asian, other Hispanic and other/mixed race categories these participants were grouped for analysis e.g., Non-Hispanic Asians, other Hispanic, other race/mixed race (Non-HA/OH/OR). Proxy-reported household income was used by the NCHS to calculate the poverty–income ratio (PIR). PIR represents the ratio of household income to poverty threshold. A ratio <1.00 indicates household income is below the poverty line threshold, whereas values >1.00 indicates income above the poverty level. Participants were grouped into the following PIR categories <1.0, 1.0–1.99, 2.0–3.49, ≥3.5 which are consistent with those used in previous analyses [13] (e.g., a ratio of 3.5 indicates that income was 350% above the poverty threshold).

### 2.3. Dietary Intake

Dietary intake was assessed via one face-to-face 24-h dietary recall. Given the age of the participants this was completed by the proxy (i.e., the parent or guardian of the child). Trained interviewers administered the dietary recall using the United States Department of Agriculture (USDA) Automated Multiple–Pass Method [18]. Dietary recalls were completed across all days of the week and throughout the year, capturing all seasons. Participants with dietary recall data that were deemed unreliable by the NCHS were excluded (*n* = 51). The What We Eat In America (WWEIA) food group classification system was used to classify beverages [19]. In this system beverages are grouped within nine major categories (“milk”, “flavoured milk”, “dairy drinks and substitutes”, “100% juice”, “diet beverages”, “sweetened beverages”, “coffee and tea”, “plain water”, “flavoured or enhanced water”, “baby beverages”, “infant formula” and “breast milk”). A complete list of all foods that fall under each beverage category is provided in Supplementary File S1.

The “milk” category includes non-flavoured whole, reduced fat, low fat and non-fat milk varieties. As per the WWEIA “milk” category definition, milk that was consumed in combination with food (e.g., cereal) has been reported here as a beverage. Of note, the majority (87%) of occurrences of milk consumed were done so as a beverage, with the remaining 13% consumed as an addition to cereal. We have also reported separately on intakes of “whole milk” and “reduced, low or non-fat milk”. All flavoured milks (whole and reduced fat varieties) fall under the category “flavoured milk”. “Dairy drink and substitutes” includes milkshakes and milk substitutes. The “100% juice” category includes 100% fruit and vegetable juices, of note in this analysis only nine children (0.4%) consumed 100% vegetable juice hence this category primarily reflects 100% fruit juice consumption. The term “100% fruit juice” will be used throughout the manuscript to describe this category. “Diet beverages” includes diet soft drinks, sports and energy drinks. “Sweetened beverages” includes sweetened soft drinks, fruit drinks, energy drinks and nutritional beverages. “Coffee and tea” includes sweetened and unsweetened, caffeinated and decaffeinated varieties. ”Plain water” includes tap water, bottled water and water consumed as an ingredient. Similar to milk, most instances (77%) of water consumed as an ingredient related to the addition of water to a beverage (e.g., water added to fruit juice) therefore water consumed as an ingredient has been categorised as a beverage. “Flavoured or enhanced water” includes flavoured or carbonated water and enhanced or fortified water. Baby beverages include products that are specifically marketed as baby beverage products within the U.S. food supply. This category consists of two sub-categories, “baby juice” and “baby water”. “Baby water” includes purified and distilled water and in this analysis these beverages were combined with the major “plain water” category. “Baby juice” includes 100% juice products and one fruit drink (40% juice), in this analysis we combined the 100% baby juices with the major “100% fruit juice” category and the fruit drink with the “sweetened beverages” category. With regards to the consumption of breast milk, the proxy only reported the frequency of feeds during the 24-h recall period and no information on the duration or quantity of feed was recorded. To quantify the amount of breast milk consumed we used a method consistent with past research [20,21] Participants who were exclusively breastfed were allocated a standard reference value of 780 mL/day of breast milk if aged 0–5.9 months and 600 mL/day of breast milk if aged 6.0–11.9 months. If the participant was partially breastfed the amount of breast milk allocated was 780 mL/day minus the total amount of “other milks (mL/day)” consumed on the day of the recall if aged 0–5.9 months; or 600 mL/day minus the total amount of “other milks (mL/day)” consumed if aged 6–11.9 months. “Other milks” included infant formula, cow’s/goat’s milk, flavoured milk or soy/rice milk. If the total daily volume of “other milks” exceeded the age specific daily reference value (i.e., 780 mL or 600 mL), the subject was allocated 89 mL of breast milk per reported feeding occasion. In children aged 12–17.9 months and 18–23.9 months, the total daily amount of breast milk was calculated as 89 mL or 59 mL, respectively, for every reported feeding occasion. The nutrient content of breast milk was obtained from the USDA NNDSR 26 [22].

To calculate nutrient intakes NHANES uses the USDA’s Food and Nutrient Database for Dietary Studies [22], which links to food composition data from the USDA National Nutrient Database for Standard Reference [19]. In this analysis we report on daily intake of water (g/day). This includes water (i.e., moisture) that is inherently found in food (e.g., water found in fruits, vegetables, meat, milk, fruit juice) as well as water consumed as a beverage. We also report on the contribution of water from food sources and beverage sources. Water from beverage sources represents the total amount of water that came from beverages as per our definition of beverages outlined above. Water from food sources represents all other sources, this includes water found in stock/broth that is coded under the WWEIA “Soup” category.

### 2.4. Statistical Analysis

Descriptive statistics (% weighting and standard error (SE) or mean and (SE)) were used to describe the proportion of participants consuming each type of beverage, mean beverage intake and mean intake of water from beverage and food sources. For beverage consumption per capita and per consumer mean intakes are reported. Results are reported by sex, age group (0–5.9 months, 6.0–11.9 months, 12.0–23.9 months), race/ethnic group and PIR level. Chi-square test was used to assess the association between sociodemographic characteristics and the consumption of each beverage type. To further examine between group differences logistic regression was used with Bonferroni adjustment for multiple comparisons. Differences in beverage intake (g/day) across sub-groups were assessed within the whole group (per capita intake) as well as within consumers only. This was done using the Wald *F*-test to assess overall group difference in mean intake (g/day) for each beverage category; where a significant group effect (*p* < 0.05) was observed an independent *T*-test with Bonferroni adjustment for multiple comparisons was used to assess differences between each sub-group. All analyses accounted for the complex survey design of NHANES and incorporated a combined 8-year dietary day one sample weight, which accounts for non-response and weekday of the dietary recall [23]. STATA/SE 14 software (StataCorp, College Station, TX, USA) was used to complete analyses.

## 3. Results

Of the 2740 participants, 51.1% were boys and the average age (SE) was 11.6 (0.2) months. The sample was comprised of 54.1% non-Hispanic white (Non-HW), 13.4% non-Hispanic Black (Non-HB), 17.8% Mexican-American (MA) and 14.7% Non-Hispanic Asian, other Hispanic and other/mixed race (Non-HA/OH/OR).

**Figure 1:**
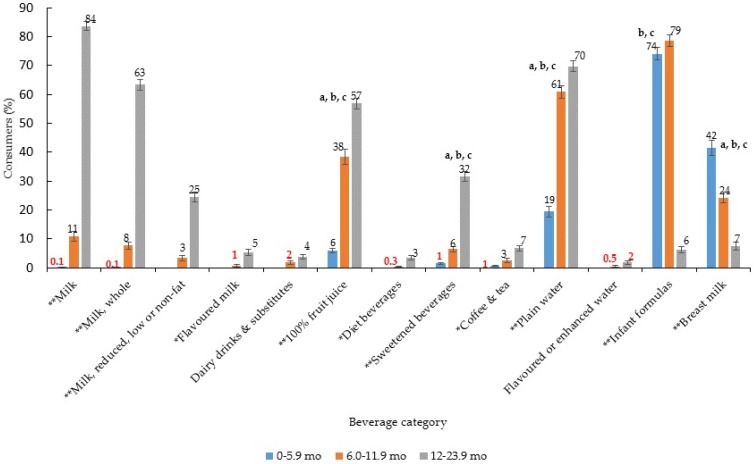
Proportion (%) of children consuming each type of beverage by age group (*n* = 2740) ^1,2,3,4,5^. ^1^ Error bars represent ± 1 SE. Those bolded in red are statistically unreliable, relative standard error ≥30% [24]; ^2^ Chi-square test to assess difference in proportions, * *p* < 0.01, ** *p* < 0.001. Where significant, logistic regression with Bonferroni adjustment for multiple comparisons was used to assess differences between sub-groups; ^a^ 0–5.9 months vs. 6.0–11.9 months *p* < 0.05; ^b^ 0–5.9 months vs. 12.0–23.9 months *p* < 0.05; ^c^ 6.0–11.9 months vs. 12.0–23.9 months *p* < 0.05; ^3^ As only *n* = 1 consumer of milk in 0–5.9 months, sub-group differences only assessed between 6.0–11.9 months and 12.0–23.9 months; ^4^ As *n* = 0 consumers in 0–5.9 months for flavoured milk, dairy drinks and substitutes, diet beverages and flavoured or enhanced water, differences in intake only assessed in 6.0–11.9 months and 12.0–23.9 months; ^5^ On the day of the 24-h dietary recall there were 2544 instances of plain water consumed across 1526 participants. *n* = 1700 (66.8%) of these instances were consumed in isolation, *n* = 650 (25.6%) were consumed as an addition to a beverage (e.g., water added to fruit juice), 7.3% instances were combined as an addition to cereal (e.g., dry, instant, rice cereal, baby food) and 0.3% were consumed with other food additions (e.g., added to fruit puree).

There were marked differences in beverage consumption patterns by age group (Figure 1, Table 1). About three quarters of infants 0–5.9 months consumed infant formula, whereas less than half consumed breast milk. Other beverages consumed at this age included plain water and 100% fruit juice. Among infants 6.0–11.9 months, infant formula remained the most commonly consumed beverage (79% consumers) and far fewer children in this age group consumed breast milk (24%). At this age a greater number of children reported consumption of 100% fruit juice (38%) and plain water (61%); and the introduction of milk (11% consumers) and sweetened beverages (6% consumers) was also apparent. Among toddlers 12.0–23.9 months the pattern continued whereby more children consumed 100% fruit juice (57%) and sweetened beverages (32%). The majority (80%) of toddlers consumed milk, with 63% consuming whole milk and 25% consuming reduced, low or non-fat varieties. Overall, intake of most beverages, was greatest in toddlers compared to younger age groups (i.e., milk, flavoured milk, dairy drinks and substitutes, 100% fruit juice, diet beverages, sweetened beverages, coffee and tea, plain water), with the exception of infant formula and breast milk where intake decreased with age (Table 1).

**Figure 2:**
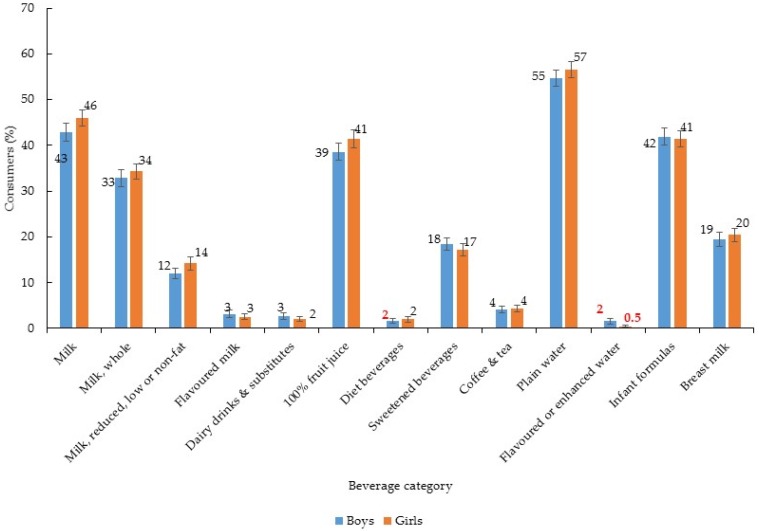
Proportion (%) of children consuming each type of beverage by sex (*n* = 2740)^1,2^; ^1^ Error bars represent ± 1 SE. Those bolded in red are statistically unreliable, relative standard error ≥30% [24]; ^2^ Chi-square test to assess difference in proportions. No significant differences between boys and girls for any beverage category.

**Figure 3:**
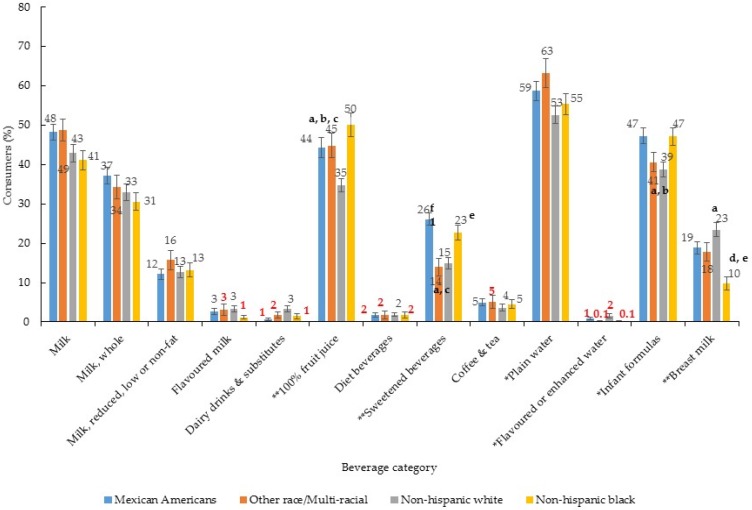
Proportion (%) of children consuming each type of beverage by race/ethnic group (*n* = 2740) ^1,2^. ^1^ Error bars represent ± 1 SE. Those bolded in red are statistically unreliable, relative standard error ≥ 30% [24]; ^2^ Chi-square test to assess difference in proportions * *p* < 0.01, ** *p* < 0.001. Where significant, logistic regression with Bonferroni adjustment for multiple comparisons was used to assess differences between sub-groups; ^a^ Non-HW vs. Non-HB *p* < 0.05; ^b^ Non-HW vs. MA *p* < 0.05; ^c^ Non-HW vs. Non-HA/OH/OR *p* < 0.05; ^d^ Non-HB vs. MA *p* < 0.05; ^e^ Non-HB vs. Non-HA/OH/OR *p* < 0.05; ^f^ MA vs. Non-HA/OH/OR *p* < 0.05.

**Figure 4:**
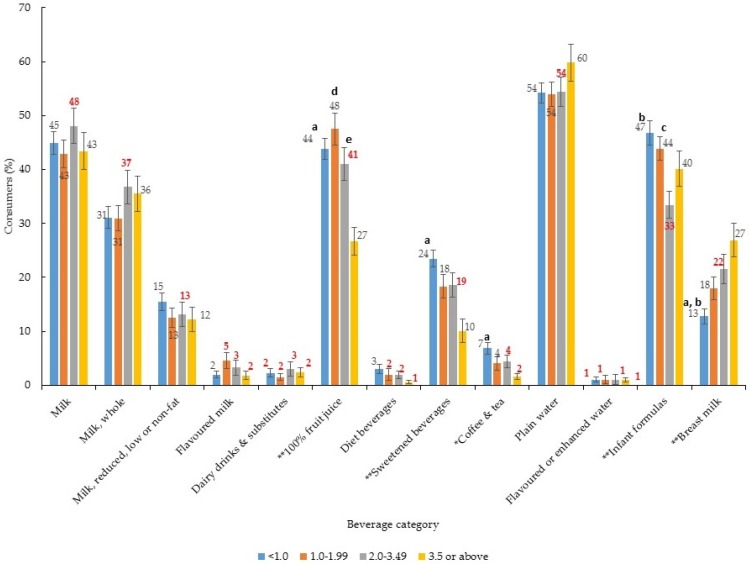
Proportion (%) of children consuming each type of beverage by poverty–income ratio (*n* = 2554) ^1,2,3^. ^1^ Error bars represent ± 1 SE. Those bolded in red are statistically unreliable, relative standard error ≥ 30% [24]; ^2^ Chi-square test to assess difference in proportions * *p* < 0.01, ** *p* < 0.001. Where significant, logistic regression with Bonferroni adjustment for multiple comparisons was used to assess differences between sub-groups; ^a^ PIR < 1.0 vs. PIR ≥ 3.5, *p* < 0.05; ^b^ PIR < 1.0 vs. PIR 2.0–3.49, *p* < 0.05; ^c^ PIR 1.00–1.99 vs. PIR 2.0–3.49, *p* < 0.05; ^d^ PIR 1.00–1.99 vs. PIR ≥ 3.5, *p* < 0.05; ^e^ PIR 2.00–3.49 vs. PIR ≥ 3.5, *p* < 0.05; ^3^ Includes those participants with data for PIR.

Beverage consumption patterns did not differ by boys and girls (Figure 2, Table 2) however there were differences by race/ethnicity (Figure 3, Table 3) and PIR (Figure 4, Table 4). Compared to Non-HW children, all other race/ethnic groups were more likely to consume 100% fruit juice (Figure 3). With regards to per capita intake of 100% fruit juice, Non-HB children consumed about 60 g/day more than Non-HW and MA children (Table 3, per capita intake). While both Non-HB and MA children were more likely to consume sweetened beverages than Non-HW children (Figure 3), the per capita amount of sweetened beverages consumed was only greater in Non-HB children compared to Non-HW (Table 3, per capita intake). Although there was an overall association between plain water consumption and race/ethnicity there were no between-group differences (Figure 3) and no difference in the per capita amount consumed (Table 3). With regards to infant formula, both MA and Non-HB children were more likely to consume this compared to Non-HW children (Figure 3) and Non-HB children were found to consume greater per capita amounts of infant formula compared to Non-HW (Table 3). Non-HB children were less likely to consume breast milk than all other race/ethnic groups (Figure 3) and average per capita intake was also lower compared to all other race/ethnic groups (Table 3, per capita intake).

The proportion of children consuming 100% fruit juice, sweetened beverages, infant formulas and breast milk differed across PIR groups (Figure 4). Children in the lowest income households (i.e., PIR < 1.0) were more likely to consume 100% fruit juice and sweetened beverages; and less likely to consume breast milk than those children living in the highest income households (i.e., PIR ≥ 3.5) (Figure 4). Compared to children from the highest income households (i.e., PIR ≥ 3.5) the per capita amount of 100% fruit juice and sweetened beverages consumed was greater among children from the lowest income households (i.e., PIR < 1.0), whereas the per capita amount of breastmilk consumed was lower (Table 4). The pattern for infant formula was less clear, whereby those children in the lowest household income group (i.e., PIR < 1.0) were more likely to be consumers of formula than those in the second highest household income groups (i.e., PIR 2.0–3.49), but there was no difference with those in the highest household income group (i.e., PIR ≥ 3.5) (Figure 4). With respects to per capita quantities consumed, intakes were greater in the lowest household income group (i.e., PIR < 1.0) compared to the top two household income groups (i.e., PIR 2.0–3.49 and PIR ≥ 3.5) (Table 4).

**Figure 5:**
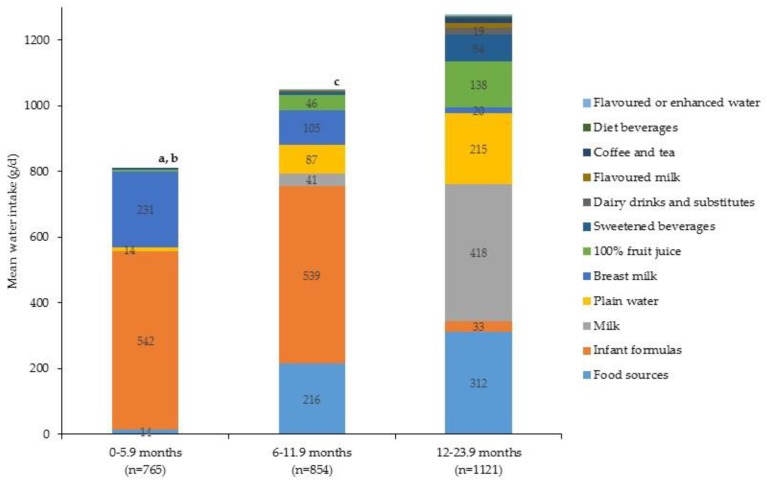
Per capita daily intake of water (g/day) from beverage and food sources by age group (*n* = 2740) ^1^. ^1^ Wald *F*-test to assess overall group difference in daily mean intake (g/day) of water *p* < 0.001; Independent *T*-test with Bonferroni adjustment for multiple comparisons was used to assess differences between each sub-group. Sub-group differences indicated by: ^a^ 0–5.9 months vs. 6.0–11.9 months *p* < 0.05; ^b^ 0–5.9 months vs. 12.0–23.9 months *p* < 0.05; ^c^ 6.0–11.9 months vs. 12.0–23.9 months *p* < 0.05.

**Figure 6:**
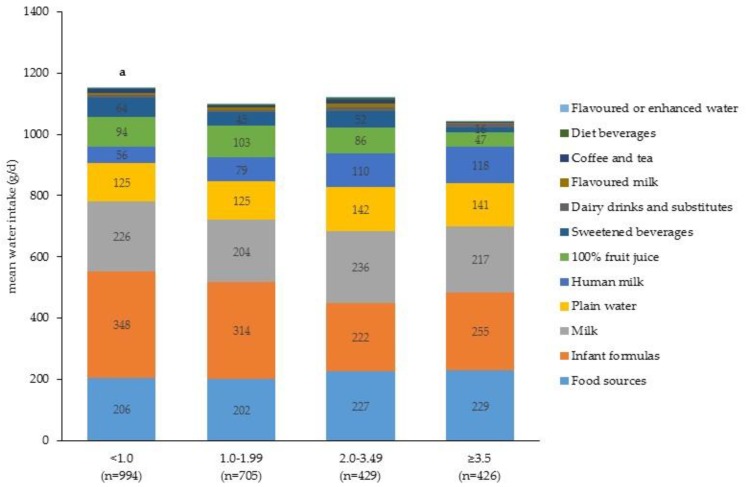
Per capita daily intake of water (g/day) from beverage and food sources by poverty–Income ratio (*n* = 2554) ^1^. ^1^ Wald *F*-test to assess overall group difference in daily mean intake (g/day) of water *p* < 0.01; Independent *T*-test with Bonferroni adjustment for multiple comparisons was used to assess differences between each sub-group. Sub-group differences indicated by: ^a^ PIR < 1.0 vs. PIR ≥ 3.5, *p* < 0.05.

Daily intake of water from beverage and food sources by age group is shown in Figure 5. Total water intake was greater among older children with intakes of 809 ± 8 g/day, 1049 ± 14 g/day and 1278 ± 19 g/day among 0–5.9 months, 6.0–11.9 months and 12–24 months, respectively (*p* < 0.001). The contribution of daily water from food and beverage sources for each age group was 0–5.9 months: 1.8% from food and 98.2% from beverages; 6.0–11.9 months: 20.6% from food and 79.4% from beverages; 12.0–23.9 months 24.4% from food and 75.6% from beverages. In infants 0–5.9 months and 6.0–11.9 months the principal dietary source of water was infant formulas, whereas for toddlers the major dietary source of water was milk, followed by food sources and plain water (Figure 5). There was no difference in daily water intake among boys and girls, 1114 ± 17 g/day and 1100 ± 15 g/day, respectively (*p* = 0.56). Likewise, the amount of water from food and beverage sources was similar with about 20% coming from food sources and 80% from beverage sources. This was the same for race/ethnicity, whereby there were no differences in total water intake and a similar contribution came from food and beverage sources. Figure 6 shows the amount of water from food and beverage sources by household income level. Total water intake did differ by household income level; intakes were higher in children from the lowest income households (1115 ± 17 g/day), compared to those children in the highest (1044 ± 23 g/day, *p* < 0.01).

## 4. Discussion

This study provides an update on beverage consumption patterns among a nationally representative sample of U.S. infants and toddlers. Whilst some positive consumption patterns were observed, such as plain milk and plain water being the most popular beverage choices among toddlers, some less desirable patterns, such as the tendency for the consumption of infant formula compared to breastmilk and the introduction of sweetened beverages were also noted, some of which were more common among lower socioeconomic and particular race/ethnic sub-groups.

During the first year of life, the focus of infant feeding relates to exclusive breastfeeding until about 6 months of age, at which point solid foods are introduced to complement breast feeding [4]. Where breastfeeding is not possible, infant formula is recommended [4]. Consistent with national reports for breastfeeding rates [25] the proportion of infants being breastfed on the day of the dietary recall decreased with age from 42% at 0–5.9 months to 24% at 6.0–11.9 months. For those aged 0–5.9 months, rates (42% consumers) are comparable to those reported in the 2008 FITS study (e.g., 42% of 4–5.9 months) yet for those aged 6.0–11.9 months consumption rates (24% consumers) are lower (e.g., 33% 6.0–11.9 months participating in 2008 FITS) [26]. We found breastfeeding differed by race/ethnicity and household income, whereby Non-HB children and those in the lowest income household groups were least likely to breastfed. Following a similar pattern Non-HB children were more likely to consume infant formulas. Barriers to breastfeeding among low income and ethnic minorities relate to lack of access to maternal information to support breastfeeding, literacy and language constraints, lack of partner support, perception that formula feeding is advantageous, early return to work and unsupportive work environments [27,28]. As breastfeeding provides a multitude of short term and long term health benefits to the child and mother [29] it is important that strategies which promote and support breastfeeding are appropriate for all race/ethnic and socioeconomic groups.

Consistent with findings from the 2008 FITS [7] we found the consumption of sweetened beverages was low among infants aged 6–11.9 months (6% consumers) however by 1–2 years almost a third (32%) of toddlers consumed these beverages. Previous reports from a cohort of Australian infants showed tracking of sugar-sweetened beverage consumption from 9–18 months of age [9]. In the US, by the age of 2–11 years 66% of children reported consumption of sugar-sweetened beverages [30]. It is not known if sweetened beverage consumption tracks over childhood however, it is apparent that for a third of U.S. toddlers consumption of these nutrient-poor beverages begins early in life.

Importantly, due to their poor nutritional quality the introduction of sweetened beverages should be discouraged [4]. Instead, healthy beverage options should be encouraged, which include breastmilk or formula during the first year of life, followed by the inclusion of water, milk and limited quantities (118–177 mL/day) of 100% fruit juice from age one [4]. Among toddlers it is reassuring to see that the majority were consumers of water and milk. Milk is the top source of both calcium and vitamin D among U.S. toddlers aged 12.0–23.9 months [1]. A recent analysis of NHANES 2009–2012 data indicated that although toddlers of this age are meeting dietary recommendations for calcium, vitamin D may be a ‘shortfall’ nutrient with 74% with intakes, calculated from food and beverage sources only (i.e., no supplements), below the Estimated Average Requirement [31].

The majority (94%) of infants aged 0–5.9 months did not consume 100% fruit juice, whereas consumption was more common among those aged 6–11.9 months (38%) and 12.0–23.9 months (57%). The American Academy of Pediatrics states that the introduction of 100% fruit juice should be avoided until the child is a toddler [4]. However, if 100% fruit juice is introduced it is advised that parents wait until the child is 6–9 months and only be provided in limited quantities of 118–177 mL/day [4] which is equivalent to 123–184 g/day (assuming a juice density of 1.04 g/mL) [32]. Similarly, intake among toddlers should also be limited to 118–177 mL/day [4]. We found the per capita quantity of 100% fruit juice was in agreement with American Academy of Pediatrics recommendations (i.e., 53 g/day and 158 g/day among 6.0–11.9 months and 12.0–23.9 months, respectively). Among consumers only intakes were higher, 163 g/day and 296 g/day, among 6.0–11.9 months and 12.0–23.9 months, respectively, indicating that among those toddlers (57%) consuming 100% fruit juice intakes may be in excess of American Academy of Pediatrics recommendations (Supplementary Table S1).

National trends indicate that among toddlers aged 1–2 years the proportion who consumed 100% fruit juice significantly increased between 1999–1994 (41%) and 2001–2006 (63%) [3]. Whilst limited quantities of 100% fruit juice (118–177 mL/day) can provide some nutritional value to the diets of children aged two years and above [33,34], there are some concerns that higher intake may relate to increased risk of dental caries and the contribution of excess calories to the diet [35]. Previous analyses within this same cohort revealed that among toddlers 100% fruit juice accounted for 6% of daily energy intake, ranking them as the 2nd source of energy [1]. Among older children aged 2–5 years participating in the 1999–2002 NHANES greater intake of 100% fruit juice was associated with higher daily energy intake [36], however there was no relationship with weight status [36]. A recent systematic review among children aged 1–18 years found no association between 100% fruit juice consumption and weight status when total energy intake was controlled for, however in those studies that did not adjust for total energy intake a positive association was reported [37]. In a prospective study of U.S. infants greater intake of 100% fruit juice at one year was associated with higher BMI *z*-scores at three and seven years of age [11]. This study also found that 100% fruit juice intake during infancy predicted 100% fruit juice intake at both time points in early childhood [11]. In children aged two years and over some studies have shown no association between 100% fruit juice consumption and weight outcomes [38,39,40] and instead shown that 100% fruit juice provides valuable short fall nutrients in the diets of children. Intake of 100% fruit juice should be monitored during early life.

Race/ethnic and socioeconomic disparities in the consumption of 100% fruit juice and sweetened beverages were apparent, whereby children of low income households and those of MA and Non-HB ethnic groups were more likely to be consumers. Similar demographic differences have been reported for 100% fruit juice consumption among U.S. infants [11] and for sweetened beverage consumption among U.S. toddlers [41] and adolescents [42]. Among children, parental modelling and home availability have been shown to be important determinants of soft drink consumption [43]. Given the dependency of young children on parents for beverage choices, such factors may be important in explaining socioeconomic differences in beverage intake. Given the well documented race/ethnic disparities that relate to childhood obesity, which begin as early as the preschool years [44], it is essential that strategies and interventions related to early life feeding practices, which include the promotion of healthy beverage options (as previously outlined above), reach all sub-groups of the population.

As expected, total daily water intake increased across age groups 809 g/day, 1049 g/day and 1278 g/day among 0–5.9 months, 6.0, 11.9 months and 12.0–23.9 months, respectively, and the contribution of water from food sources also increased (e.g., 1.8%, 21.8% and 24.4%, respectively), reflecting the shift to complementary feeding practices. The only demographic variable that was related to total water intake, was household income, with the highest intakes reported in those children from the lowest income households. This may be explained by overall greater intakes of sweetened beverages and infant formula among this group, both of which have a high water content. The Adequate Intake (AI) for total water for infants 0–6 months is 700 g/day [12]. This recommendation is based on the average amount of breastmilk consumed and its corresponding water content. For 7–12 month olds the AI increases to 800 g/day, accounting for water from complementary foods and beverages and for age 1–3 years the AI is 1300 g/day [12]. Thus, reported group average intakes of total water meet or exceed the recommended AIs for each age group in our study, which indicates that the prevalence of inadequate water intake is likely to be low in U.S. infants and toddlers.

This study has a number of limitations. Firstly, the use of proxy-reported 24-h dietary recalls to measure food intake is subject to reporting errors. Furthermore, information on the amount of breast milk consumed by breastfed infants was not available and instead established methods [1,20,21] were used to estimate breast milk intake. We grouped beverages according to the WWEIA beverage categories and did not separate beverages which may have been consumed in combination with a food (e.g., milk and cereal). This is likely to have minimal impact on the results as the consumption of beverages with food was relatively infrequent. The race/ethnic group ‘Non-Hispanic Asians’ was only oversampled since 2011–2012, as such the low numbers within this group did not allow for comparisons and instead were combined with the Other Hispanic and Other race/mixed-race groups. Strengths of this analysis include the use of data from NHANES, which is a nationally representative study which includes the collection of dietary data throughout all seasons of the year. Furthermore, the combination of four survey cycles provided an adequate sample size which enabled comparison of beverage intake across sociodemographic groups.

## 5. Conclusions

In conclusion, the findings from this study indicate that important sociodemographic differences in beverage consumption patterns are apparent from early on in life. Strategies that seek to improve the quality of beverages consumed by U.S. infants and toddlers are needed, with a focus on encouraging uptake and continuation of breastfeeding during the first year of life along with beverage choices which are in line with dietary recommendations for toddlers. Additional longitudinal studies are needed to monitor beverage consumption patterns during early life.

## Figures and Tables

**Table 1:** Beverage consumption by age group (*n* = 2740).

Beverage Category	Per Capita Mean Intake ± SE (g/Day)				Per Consumer Mean Intake ± SE (g/Day)			
	0–5.9 Months	6.0–11.9 Months	12.0–23.9 Months	*p*-Value ^1^	0–5.9 Months	6.0–11.9 Months	12.0–23.9 Months	*p*-Value ^1^
Milk ^2^	0 0	46 ± 8	472 ± 14.4	<0.001	7.6 ^††^	426 ± 41	565 ± 14	<0.001
Milk, whole ^2^	0 ± 0	35 ± 7	363 ± 12.7	<0.001	7.6 ^††^	460 ± 48	572 ± 15	0.03
Milk, reduced, low or non-fat ^3^	0	11 ± 4	109 ± 9.1	<0.001	n/a	325 ± 76	446 ± 26	0.14 ^3^
Flavoured milk ^3^	0	1 ± 0.3 ^†^	20 ± 4.8	<0.001	n/a	95 ± 29 ^†^	363 ± 59	<0.001
Dairy drinks & substitutes ^3^	0	4 ± 2 ^†^	21 ± 4.8	0.03	n/a	234 ± 105 ^†,c^	542 ± 79	0.04
100% fruit juice	6 ± 1 ^a,b^	53 ± 4 ^c^	158 ± 10.0	<0.001	99 ± 12 ^a,b^	138 ± 7 ^c^	278 ± 13	<0.001
Diet beverages ^3^	0	0.7 ± 0.4 ^†^	6 ± 1.3	<0.001	n/a	22 ± 17 ^†^	183 ± 24	<0.001
Sweetened beverages	2 ± 1 ^†,a,b^	10 ± 2 ^†,c^	94 ± 8.0	<0.001	155 ± 74 ^†^	163 ± 23 ^c^	296 ± 20	<0.001
Coffee & tea	1 ± 0.5 ^†,b^	2 ± 1 ^†,c^	15 ± 3.6	<0.001	132 ± 33	77 ± 14 ^c^	223 ± 44	0.01
Plain water	15 ± 2 ^a,b^	87 ± 6 ^c^	215 ± 9.6	<0.001	75 ± 10 ^a,b^	143 ± 10 ^c^	308 ± 10	<0.001
Flavoured or enhanced water ^3^	0	0 ± 0.4 ^†^	4 ± 2.1 ^†^	0.07	n/a	87 ± 16	236 ± 52	0.01
Infant formulas	618 ± 23 ^b^	615 ± 20 ^c^	38 ± 6.8	<0.001	834 ± 17 ^b^	783 ± 15 ^c^	591 ± 48	<0.001
Breast milk	264 ± 18 ^a,b^	119 ± 11 ^c^	23 ± 4.0	<0.001	635 ± 17 ^a,b^	492 ± 15 ^c^	304 ± 33	<0.001
Total beverage intake	905 ± 10 ^b^	938 ± 13 ^c^	1065 ± 18.3	<0.001	n/a	n/a	n/a	

^1^ Wald *F*-test to assess overall group difference in per capita or consumer mean intake (g/day); where significant group effect was found, Independent *T*-test with Bonferroni adjustment for multiple comparisons was used to assess differences between each sub-group. Sub-group differences indicated by: ^a^ 0–5.9 months vs. 6.0–11.9 months *p* < 0.05; ^b^ 0–5.9 months vs. 12.0–23.9 months *p* < 0.05; ^c^ 6.0–11.9 months vs. 12.0–23.9 months *p* < 0.05; ^2^ As only *n* = 1 consumer in 0–5.9 months, differences in intake only assessed in 6.0–11.9 months and 12.0–23.9 months; ^3^ As *n* = 0 consumers in 0–5.9 months, differences in intake only assessed in 6.0–11.9 months and 12.0–23.9 months; ^†^ Data are statistically unreliable, relative standard error ≥30% [24]; ^††^ No SE calculated as only 1 consumer; n/a represent not applicable as not possible to calculate mean intake as there were no consumers of the beverage category. Nonsensical to calculate total beverage intake as the number of consumers varies across each beverage category.

**Table 2:** Beverage consumption by sex (*n* = 2740).

Beverage Category	Per Capita Mean Intake ± SE (g/Day)			Per Consumer Mean Intake ± SE (g/Day)		
	Boys	Girls	*p*-Value ^1^	Boys	Girls	*p*-Value ^1^
Milk	245 ± 13.2	249 ± 13.2	0.82	571 ± 18	541 ± 16	0.19
Milk, whole	188 ± 11.5	192 ± 11.3	0.83	572 ± 20	558 ± 17	0.57
Milk, reduced, low or non-fat	57 ± 6.7	58 ± 7.2	0.94	475 ± 37	405 ± 30	0.13
Flavoured milk	10 ± 4.1 ^†^	10 ± 2.8	0.96	314 ± 88	383 ± 64	0.53
Dairy drinks & substitutes	11 ± 3.2 ^†^	12 ± 3.8 ^†^	0.72	392 ± 81	598 ± 98	0.13
100% fruit juice	91 ± 6.9	97 ± 7.7	0.57	236 ± 13	235 ± 13	0.95
Diet beverages	2 ± 0.7 ^†^	4 ± 1.4 ^†^	0.17	122 ± 29	216 ± 44	0.16
Sweetened beverages	55 ± 6.3	44 ± 4.4	0.15	302 ± 25	258 ± 24	0.23
Coffee & tea	9 ± 3.3 ^†^	7 ± 1.7	0.66	219 ± 73	173 ± 24	0.56
Plain water	132 ± 8.2	136 ± 7.0	0.70	240 ± 11 ^†^	240 ± 11	0.98
Flavoured or enhanced water	3 ± 2.0 ^†^	1 ± 0.6 ^†^	0.23	224 ± 58	194 ± 76 ^†^	0.76
Infant formulas	335 ± 15.9	322 ± 14.8	0.51	800 ± 17	778 ± 17	0.36
Breast milk	102 ± 9.0	108 ± 8.1	0.69	526 ± 20	526 ± 17	0.99
Total beverage intake	996 ± 13.6	991 ± 14.1	0.79	n/a	n/a	

^1^ Independent *T*-test to assess difference in per capita or consumer mean intake (g/day) by sex; ^†^ Data are statistically unreliable, relative standard error ≥30% [24]; n/a represent not applicable as nonsensical to calculate total beverage intake as the number of consumers varies across each beverage category.

**Table 3:** Beverage consumption by race/ethnic group (*n* = 2740).

Beverage Category	Per Capita Mean Intake ± SE (g/Day)					Per Consumer Mean Intake ± SE (g/Day)				
	Non-HW	Non-HB	MA	Non-HA/OH/OR	*p*-Value ^1^	Non-HW	Non-HB	MA	Non-HA/OH/OR	*p*-Value ^1^
Milk	246 ± 15	210 ± 18	271 ± 15	255 ± 19	0.08	574 ± 20	511 ± 26	563 ± 24	522 ± 30	0.08
Milk, whole	189 ± 13	167 ± 17	210 ± 16	190 ± 21	0.33	573 ± 22	546 ± 36	566 ± 30	554 ± 36	0.86
Milk, reduced, low or non-fat	57 ± 8	43 ± 7	61 ± 8	65 ± 12	0.34	453 ± 38	329 ± 39 ^b^	502 ± 40	412 ± 45	0.02
Flavoured milk	12 ± 4 ^†^	4 ± 2 ^†^	11 ± 4 ^†^	7 ± 4 ^†^	0.16	357 ± 82	344 ± 88	396 ± 119 ^†^	238 ± 29	0.28
Dairy drinks & substitutes	16 ± 4	5 ± 3 ^†^	3 ± 2 ^†^	10 ± 6 ^†^	0.06	491 ± 78	340 ± 82	404 ± 184 ^†^	559 ± 220 ^†^	0.54
100% fruit juice	80 ± 7 ^a^	146 ± 17 ^b^	84 ± 7	111 ± 13	0.001	230 ± 14	292 ± 25 ^b^	189 ± 10	249 ± 23	0.01
Diet beverages	3 ± 1	2 ± 1 ^†^	3 ± 1 ^†^	2 ± 2 ^†^	0.85	197 ± 41	135 ± 38	160 ± 53 ^†^	141 ± 49 ^†^	0.63
Sweetened beverages	38 ± 7 ^a^	73 ± 8	69 ± 11	50 ± 14	0.001	256 ± 37	320 ± 23	262 ± 37	361 ± 76	0.51
Coffee & tea	9.0 ± 3	11 ± 3 ^†^	7 ± 2	6 ± 2 ^†^	0.62	236 ± 71	233 ± 55	149 ± 26	118 ± 23	0.02
Plain water	102 ± 7	122 ± 10	108 ± 7	129 ± 12	0.52	243 ± 12	249 ± 14	208 ± 11	222 ± 14	0.05
Flavoured or enhanced water	4 ± 2	0.3 ± 0.3 ^†^	2 ± 0.8 ^†^	0.1 ± 0.1 ^†^	0.11	226 ± 63	237 ^††^	186 ± 41 ^e^	46 ^††^	0.001
Infant formulas	296 ± 17 ^a^	404 ± 25	363 ± 15	338 ± 21	0.003	765 ± 20 ^a^	859 ± 27 ^b^	767 ± 20	832 ± 18	<0.01
Breast milk	126 ± 9 ^a^	48 ± 9 ^b,c^	91 ± 9	99 ± 15	<0.001	535 ± 18	486 ± 31	483 ± 21	558 ± 27	0.19
Total beverage intake	932 ± 17 ^a^	1025 ± 22	1010 ± 18	1008 ± 22	0.01	n/a	n/a	n/a	n/a	

^1^ Wald *F*-test to assess overall group difference in per capita or consumer mean intake (g/day); where significant group effect was found, Independent *T*-test with Bonferroni adjustment for multiple comparisons was used to assess differences between each sub-group. Sub-group differences indicated by: ^a^ Non-HW vs. Non-HB *p* < 0.05; ^b^ Non-HB vs. MA *p* < 0.05; ^c^ Non-HB vs. Non-HA/OH/OR *p* < 0.05; ^d^ Non-HW vs. MA *p* < 0.05; ^e^ MA vs. Non-HA/OH/OR *p* < 0.05; ^†^ Data are statistically unreliable, relative standard error ≥30% [24]; ^††^ No SE calculated as only 1 consumer; n/a represent not applicable as nonsensical to calculate total beverage intake as the number of consumers varies across each beverage category.

**Table 4:** Beverage consumption by poverty–income ratio (*n* = 2554) ^1,2^.

Beverage Category	Per Capita Mean Intake ± SE (g/Day)					Per Consumer Mean Intake ± SE (g/Day)				
	PIR < 1.0	PIR1.0–1.99	PIR 2.0–3.49	PIR ≥ 3.5	*p*-Value ^2^	PIR < 1.0	PIR1.0–1.99	PIR2.0–3.49	PIR ≥ 3.5	*p*-Value ^2^
Milk	256 ± 16	231 ± 16	267 ± 25	245 ± 27	0.62	569 ± 22	538 ± 25	556 ± 37	565 ± 30	0.86
Milk, whole	184 ± 12	177 ± 16	200 ± 21	202 ± 24	0.77	592 ± 23	570 ± 29	543 ± 39	569 ± 29	0.70
Milk, reduced, low or non-fat	71 ± 11	54 ± 8	68 ± 16	43 ± 9	0.21	462 ± 50	434 ± 41	513 ± 56	353 ± 42	0.14
Flavoured milk	7 ± 3	11 ± 3 ^†^	16 ± 9 ^†^	7 ± 4 ^†^	0.65	351 ± 105 ^†^	240 ± 43	483 ± 141	368 ± 133 ^†^	0.31
Dairy drinks & substitutes	12 ± 5 ^†^	7 ± 4 ^†^	15 ± 7 ^†^	12 ± 6 ^†^	0.73	531 ± 95	492 ± 89	489 ± 133	485 ± 155 ^†^	0.99
100% fruit juice	108 ± 9 ^a^	118 ± 12 ^c^	99 ± 13 ^d^	54 ± 8	0.001	246 ± 15	249 ± 19	240 ± 25	202 ± 23	0.46
Diet beverages	4 ± 1	3 ± 2 ^†^	5 ± 2 ^†^	2 ± 1 ^†^	0.49	124 ± 29 ^a^	170 ± 59 ^†^	233 ± 63	301 ± 47	0.02
Sweetened beverages	71 ± 8 ^a^	51 ± 10 ^c^	57 ± 16	18 ± 5	<0.001	304 ± 23 ^a^	275 ± 43	305 ± 72	178 ± 26	<0.01
Coffee & tea	13 ± 3 ^a^	5 ± 2 ^†^	14 ± 8 ^†^	2 ± 1 ^†^	0.004	182 ± 29	128 ± 38 ^†^	308 ± 138 ^†^	119 ± 27 ^†^	0.32
Plain water	109 ± 7	96 ± 7	121 ± 12	116 ± 11	0.50	230 ± 11	216 ± 13 ^e^	258 ± 19	234 ± 17	0.33
Flavoured or enhanced water	2 ± 1 ^†^	2.0 ± 1 ^†^	4 ± 4 ^†^	2 ± 0.9 ^†^	0.93	170 ± 62 ^†^	147 ± 34	414 ± 21 ^d^	169 ± 55 ^†^	0.001
Infant formulas	397 ± 21 ^a,b^	359 ± 20 ^e^	252 ± 19	291 ± 29	<0.001	848 ± 20 ^a,b^	817 ± 17 ^c^	755 ± 28	726 ± 31	<0.01
Breast milk	64 ± 7.0 ^a,b^	90 ± 12	126 ± 15	135 ± 17	<0.001	505 ± 26	504 ± 30	584 ± 25	503 ± 27	0.09
Total beverage intake	1042 ± 16 ^a^	973 ± 18 ^c^	975 ± 33	834 ± 19	<0.001	n/a	n/a	n/a	n/a	

^1^ Includes those participants with data for poverty–income ratio; ^2^ Wald *F*-test to assess overall group difference in per capita or consumer mean intake (g/day); where significant group effect was found, Independent *T*-test with Bonferroni adjustment for multiple comparisons was used to assess differences between each sub-group. Sub-group differences indicated by: ^a^ PIR <1.0 vs. PIR ≥ 3.5, *p* < 0.05; ^b^ PIR < 1.0 vs. PIR 2.0–3.49, *p* < 0.05; ^c^ PIR 1.00–1.99 vs. PIR ≥ 3.5, *p* < 0.05; ^d^ PIR 2.00–3.49 vs. PIR ≥ 3.5, *p* < 0.05; ^e^ PIR 1.00–1.99 vs. PIR 2.0–3.49, *p* < 0.05; ^†^ Data are statistically unreliable, relative standard error ≥30% [24]; ^††^ No SE calculated as only 1 consumer; n/a represent not applicable as nonsensical to calculate total beverage intake as the number of consumers varies across each beverage category.

## Supplementary Materials

The following are available online at http://www.mdpi.com/2072-6643/9/3/264/s1, File S1: WWEIA Beverage List, Table S1: Overview of American Academy of Pediatrics (AAP) Infant and Toddler Feeding Guidelines [4] and intakes of 100% juice and sweetened beverages among U.S infants and toddlers aged 0–23.9 months.
