# Supplementary material for: Beverage Consumption among U.S. Children Aged 0–24 Months: National Health and Nutrition Examination Survey (NHANES)
Source: Nutrients. 2017 Mar 13;9(3):264. doi: 10.3390/nu9030264 (PMC5372927; doi:10.3390/nu9030264)
Supplement: Supplementary file 1 [file nutrients-09-00264-s001.zip › nutrients-176581 Supplementary/Supplementary Table S1.docx]

**Supplementary Materials: Beverage Consumption among U.S. Children Aged 0–24 Months: National Health and Nutrition Examination Survey (NHANES)**

Carley A. Grime, Ewa A. Szymlek-Gay and Theresa A. Nicklas

4. Discussion

The majority (94%) of infants aged 0–5.9 months did not consume 100% fruit juice, whereas consumption was more common among those aged 6–11.9 months (38%) and 12.0–23.9 months (57%). The American Academy of Pediatrics states that the introduction of 100% fruit juice should be avoided until the child is a toddler [[1](#_ENREF_4)]. However, if 100% fruit juice is introduced it is advised that parents wait until the child is 6–9 months and only be provided in limited quantities of 118–177 mL/day [[1](#_ENREF_4)] which is equivalent to 123–184 g/day (assuming a juice density of 1.04 g/mL) [[2](#_ENREF_44)]. Similarly, intake among toddlers should also be limited to 118–177 mL/day [[1](#_ENREF_4)]. We found the per capita quantity of 100% fruit juice was in agreement with American Academy of Pediatrics recommendations (i.e., 53 g/day and 158 g/day among 6.0–11.9 months and 12.0–23.9 months, respectively). Among consumers only intakes were higher, 163 g/day and 296 g/day, among 6.0–11.9 months and 12.0–23.9 months, respectively, indicating that among those toddlers (57%) consuming 100% fruit juice intakes may be in excess of American Academy of Pediatrics recommendations (Supplementary Table S1).

**Table S1. Overview of American Academy of Pediatrics (AAP) Infant and Toddler Feeding Guidelines [**1**] and intakes of 100% juice and sweetened beverages among U.S infants and toddlers aged 0-23.9 months**

| **Age group** | **AAP Guideline** | **Mean 100% juice intake (g/day)** | | **Mean sweetened beverage intake (g/day)** | |
| --- | --- | --- | --- | --- | --- |
|  |  | **Per**  **capita** | **Per consumer** | **Per**  **capita** | **Per consumer** |
| 0-1 year | - Babies should drink breastmilk or formula for the first year of life | 6 | 99 | 2 | 155 |
| 6-12 months | - Babies should drink breastmilk or formula for the first year of life  - Try to avoid introducing juice until child is a toddler. If juice is introduced, wait until 6-9 months and limit consumption to 4-6 ounces (i.e. 118-117 ml/day)  - Avoid introduction of sugar-sweetened beverages | 53 | 138 | 10 | 163 |
| 1-2 years | - Choose milk or water for your child's beverage  - Limit consumption of juice to 4-6 ounces (i.e. 118-117 ml/day)  - Avoid introduction of sugar-sweetened beverages | 158 | 278 | 94 | 296 |

**References**

1. American Academy of Pediatrics. Food and Feeding—Age Specific Content. Available online: https://www.aap.org/en-us/advocacy-and-policy/aap-health-initiatives/HALF-Implementation-Guide/Age-Specific-Content/Pages/Age-Specific-Content.aspx (accessed on 6 October 2016).

2. Food and Agriculture Organization of the United Nations (FAO). FAO/INFOODS Databases Density Database Version 2.0. Available online: http://www.fao.org/docrep/017/ap815e/ap815e.pdf (accessed on 16 December 2017).
